# Supplementary figures and images for: Deciphering lentiviral Vpr/Vpx determinants required for HUSH and SAMHD1 antagonism highlights the molecular plasticity of these evolutionary conflicts
Source: J Virol. 2025 Apr 22;99(5):e00198-25. doi: 10.1128/jvi.00198-25 (PMC12090772; doi:10.1128/jvi.00198-25)

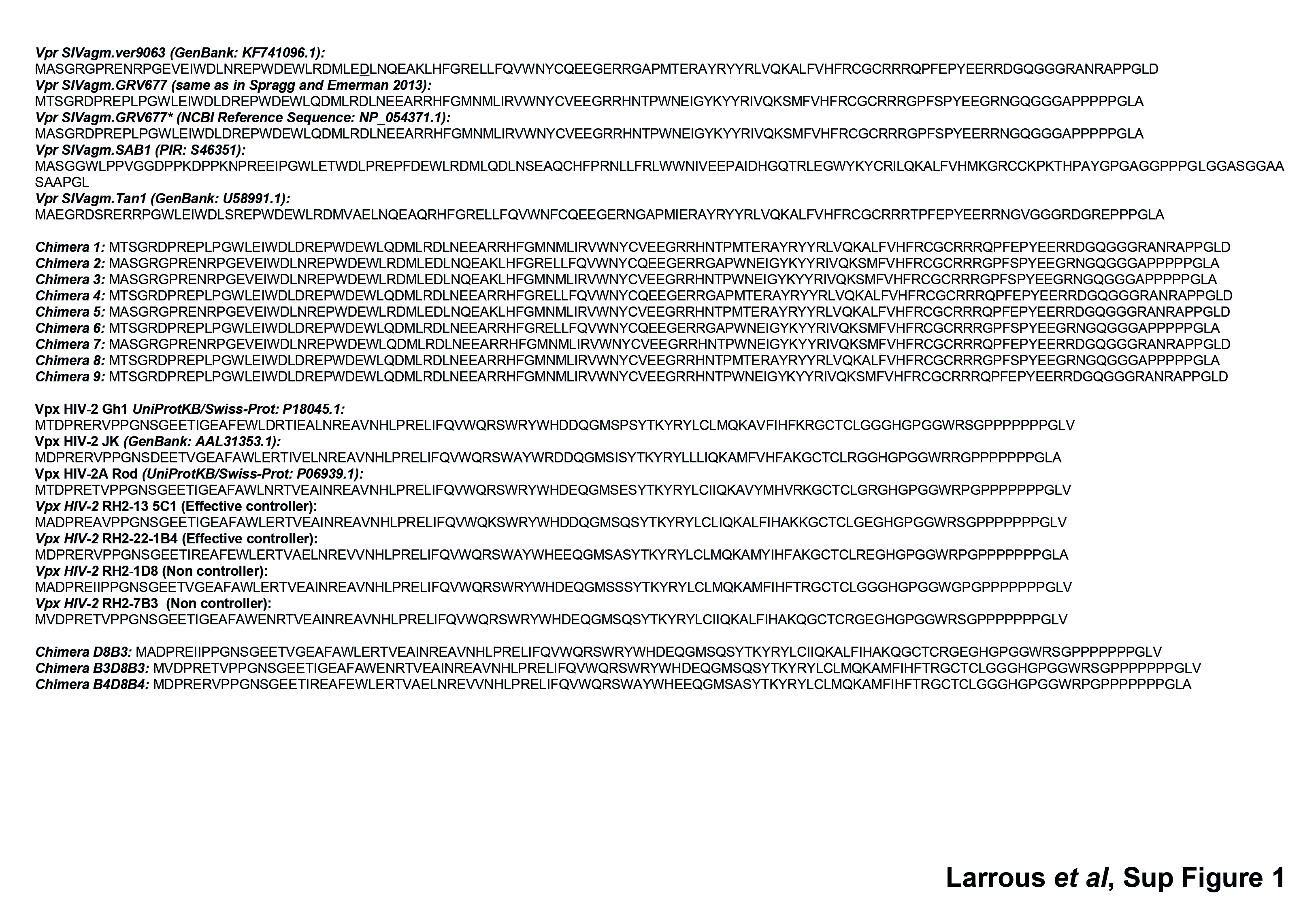

Supplement: Figure S1 — Amino acid sequences of SIVagm Vpr proteins, HIV-2 Vpx proteins, and chimera. [file jvi.00198-25-s0001.tif]
